# Adaptation of ACMG/AMP Guidelines for Clinical Classification of *BMPR2* Variants in Pulmonary Arterial Hypertension Resolves Variants of Unclear Pathogenicity in ClinVar

**DOI:** 10.1155/humu/2475635

**Published:** 2025-07-06

**Authors:** Christina A. Eichstaedt, Gabriel Maldonado-Velez, Rajiv D. Machado, Stefan Gräf, Dennis Dooijes, Srimmitha Balachandar, Florence Coulet, Kristina Day, Melanie Eyries, Daniela Macaya, Memoona Shaukat, Laura Southgate, Jair Tenorio-Castano, Wendy K. Chung, Carrie L. Welch, Micheala A. Aldred

**Affiliations:** ^1^Center for Pulmonary Hypertension, Thoraxklinik-Heidelberg gGmbH, Heidelberg University Hospital and Translational Lung Research Center (TLRC), German Center for Lung Research (DZL), Heidelberg, Baden-Württemberg, Germany; ^2^Laboratory for Molecular Genetic Diagnostics, Institute of Human Genetics, Heidelberg University, Heidelberg, Baden-Württemberg, Germany; ^3^Division of Pulmonary, Critical Care, Sleep and Occupational Medicine, Department of Medicine, Indiana University School of Medicine, Indianapolis, Indiana, USA; ^4^School of Health & Medical Sciences, City St George's, University of London, London, UK; ^5^Department of Medicine, School of Clinical Medicine, University of Cambridge, Victor Phillip Dahdaleh Heart & Lung Research Institute, Cambridge, UK; ^6^Department of Genetics, University Medical Centre Utrecht, Utrecht University, Utrecht, the Netherlands; ^7^Saint-Antoine Research Centre, Sorbonne University, Paris, France; ^8^Genetics Department, AP-HP Sorbonne University, Paris, France; ^9^GeneDx Inc., Gaithersburg, Maryland, USA; ^10^Institute of Medical and Molecular Genetics (INGEMM), La Paz University Hospital, IDiPAZ, Autonoma University of Madrid, Madrid, Spain; ^11^Biomedical Research Network Centre for Rare Diseases (CIBERER), Carlos III Health Institute, Madrid, Spain; ^12^ITHACA, European Reference Network, Brussels, Belgium; ^13^Division of Genetics and Genomics, Department of Pediatrics, Boston Children's Hospital and Harvard Medical School, Boston, Massachusetts, USA

**Keywords:** ACMG/AMP guidelines, BMPR2, ClinVar, pulmonary arterial hypertension, variant interpretation

## Abstract

Pulmonary arterial hypertension (PAH) is a rare disease that can be caused by pathogenic variants, most frequently in the bone Morphogenetic Protein Receptor Type 2 (*BMPR2*) gene. We formed a ClinGen variant curation expert panel to devise guidelines for the clinical interpretation of *BMPR2* variants identified in PAH patients. The general ACMG/AMP variant classification criteria were refined for PAH and adapted to *BMPR2* following ClinGen procedures. Subsequently, these specifications were tested independently by three members of the curation expert panel on 28 representative *BMPR2* variants selected from ClinVar and then presented and discussed in the plenum. Application of the final *BMPR2* variant specifications resolved six of nine variants (66%) where multiple ClinVar classifications included a variant of uncertain significance, with all six being reclassified as Benign or Likely Benign. Four splice site variants underwent clinically consequential reclassification based on the presence or absence of supporting mRNA splicing data. These variant specifications provide an international framework and a valuable tool for *BMPR2* variant classification that can be applied to increase confidence and consistency in *BMPR2* interpretation for diagnostic laboratories, clinical providers, and patients.

## 1. Introduction

Pulmonary arterial hypertension (PAH; OMIM #178600) is a rare disease with a prevalence of 15–50 cases per one million individuals [1, 2]. Symptoms of PAH are nonspecific including shortness of breath particularly during exertion, palpitations, and, in advanced cases, edema, chest pain, and dyspnea at rest. While noninvasive assessments such as echocardiography, electrocardiography, laboratory tests, pulmonary function tests, and cardiopulmonary exercise testing can support a diagnosis of PAH, the definitive diagnosis is made by right heart catheterization. PAH is hemodynamically defined by an elevated mean pulmonary artery pressure > 20 mmHg, pulmonary vascular resistance > 2 Wood units, and a pulmonary artery wedge pressure ≤ 15 mmHg [2]. PAH is currently classified as idiopathic (IPAH), heritable (HPAH), or associated with other diseases or exposures, including connective tissue disease, congenital heart disease, HIV infection, or drugs and toxins. By definition, patients with HPAH have a positive family history and/or carry a pathogenic variant in a definitive PAH risk gene. Thus, apparently idiopathic patients may be reclassified as HPAH following genetic analysis.

The first gene identified as causative for PAH encodes the Bone Morphogenetic Protein Receptor Type 2 (*BMPR2*; HGNC: 1078) [3, 4], and it remains the gene with the highest frequency of Pathogenic variants in HPAH patients [5–7]. BMPR2 is a cell surface receptor and part of the Transforming Growth Factor *β* superfamily responsible for vascular homeostasis of cell proliferation and apoptosis. Heterozygous Pathogenic or Likely Pathogenic (LP) *BMPR2* variants lead to PAH in an autosomal dominant manner with reduced penetrance of approximately 15%–40% and show sexual dimorphism, with higher penetrance in females [8].

Subsequently, several additional genes have been implicated in the etiology of IPAH and HPAH, especially with the advent of next-generation sequencing of large cohorts [9–11]. A recent study carried out by our Clinical Genome Resource (ClinGen) pulmonary hypertension gene curation expert panel (PH GCEP) thoroughly characterized 27 genes and classified 12 of them to have a definitive gene–disease relationship with IPAH/HPAH (*ACVRL1*, *ATP13A3*, *BMPR2*, *CAV1*, *EIF2AK4*, *ENG*, *GDF2*, *KCNK3*, *KDR*, *SMAD9*, *SOX17*, and *TBX4*), three to have moderate evidence (*ABCC8*, *GGCX*, and *TET2*), six with limited evidence (*AQP1*, *BMP10*, *FBLN2*, *KLF2*, *KLK1*, and *PDGFD*), and one to have no evidence for the relationship with PAH (*TOPBP1*) [12]. Notably, four genes in the *BMPR2* pathway (*BMPR1A*, *BMPR1B*, *SMAD1*, and *SMAD4*) were disputed as causative PAH genes [12].

ClinGen, funded by the National Institutes of Health (NIH), serves as a central resource with information on the clinical significance of genes and variants [13]. Curation of genes and variants focuses on gene disease validity, variant pathogenicity, dosage sensitivity, and clinical actionability. ClinGen also offers the opportunity to form expert panels to curate not only genes but also variants [14]. To this end, following our gene curation efforts, we founded a PH variant curation expert panel (VCEP) to adapt the American College of Medical Genetics and Genomics (ACMG)/Association of Molecular Pathology (AMP) guidelines with a focus on *BMPR2*. We modified the general variant curation guidelines by the ACMG/AMP framework [15] for *BMPR2* variants specific to HPAH and/or IPAH. Since a significant number of *BMPR2* variants have an unclear impact on gene expression or function we aimed to resolve conflicting classifications to improve the interpretation of genetic test results. We tested our refined specification criteria on a pilot subset of 28 representative *BMPR2* variants, the results of which are described herein.

## 2. Methods

**Figure 1:**
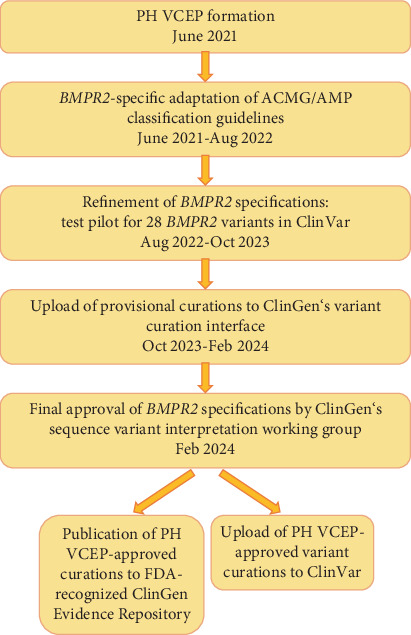
Flow chart of pulmonary hypertension variant curation expert panel formation and activities.

Within the framework of our Genetics Task Force of the International Consortium for Genetic Studies in PAH (PAH-ICON, https://pahicon.com/), we founded a ClinGen Pulmonary Hypertension VCEP with 16 members from nine institutions across the United States and Europe, with representation from six countries (https://www.clinicalgenome.org/affiliation/50071/). Members included a chairperson (WKC), scientific lead/coordinator (CLW), expert reviewers, and biocurators. Expert reviewer and biocurator roles were assigned based on members' experience, but most members had dual roles. Seven members hold professional certifications in clinical genetics and/or regularly use ACMG/AMP guidelines to classify variants for clinical laboratory case sign-out. Most members had a history of ClinGen activities including curation of PAH gene relationships [12]. All members received additional training in the ClinGen variant curation standard operating procedure V3, the 2015 ACMG/AMP guidelines, the ClinGen sequence variant interpretation working group recommendations for applying ACMG/AMP criteria, sequence variant literature searches, and use of ClinGen's variant curation interface (VCI) [16]. We followed the ClinGen four-step process (protocol Version 9) to obtain VCEP approval (February 2024) with resulting variant classifications meeting US Food and Drug Administration (FDA) recognition. A flow chart for the VCEP formation and activities is shown in Figure 1.

We limited the scope of this work to *BMPR2* variants because of the large number of loss-of-function variants, recurrent variants, and the relatively large body of experimental evidence defining critical domains/residues and functional effects of individual variants compared to other PAH risk genes [12]. Monthly virtual meetings were held over a 14-month period (June 2021 to August 2022) to develop variant classification rules for *BMPR2* based on the ACMG/AMP guidelines (Figure 1). We then tested our adapted specifications on a pilot group of 28 variants, representing all ACMG classes, including those with conflicting interpretations of pathogenicity. The selected variants included insertions, deletions, 5⁣′-untranslated region, missense, splice, nonsense, and synonymous variants. Only *BMPR2* variants identified in nonsyndromic HPAH and/or IPAH cases were considered for pilot curation. Thus, one additional variant (c.319T>C p.Ser107Pro) was removed from the pilot curation list as it had been described in a proband with congenital heart disease–associated PAH and was therefore outside the scope of this work. The *BMPR2* MANE Select transcript with accession number NM_001204.7 was used as the reference for all variant curations.

We assigned six variants to each curator and teams of three curators per variant. Provisional classifications were presented and discussed at monthly meetings over another 14-month period (August 2022 to October 2023). Modifications of our *BMPR2* criteria specifications were made over this period and following guidance of the ClinGen sequence variant interpretation working group. The specifications are available in the ClinGen Criteria Specification Registry (https://cspec.genome.network/cspec/ui/svi/doc/GN125). Final curations were uploaded to the VCI. Following the final PH VCEP approval, variant curations were published to the ClinGen Evidence Repository and submitted to ClinVar (https://www.ncbi.nlm.nih.gov/clinvar/?term=bmpr2%255Bgene%255D%26redir=gene). All members disclosed conflicts of interest, and the curation assignments were mindful of conflicts. Our ACMG/AMP specifications are updated periodically. To find the most current information, please visit https://cspec.genome.network.

## 3. Results

### 3.1. Development of *BMPR2*-Specific Variant Curation Criteria

#### 3.1.1. Population Frequency-Based Criteria: PM2, BA1, BS1, and BS2

The population frequency criteria evaluation includes four criteria (Table 1). Based on our *BMPR2* variant specifications, we applied PM2 as supporting when the variant was absent from the gnomAD V2.1.1 (controls) and V3.1.2 (controls/biobanks) populations or had an allele frequency below 0.01%. For this, we considered the ancestral subgroup with the highest frequency and at least 1000 allele counts. The 0.01% threshold for allele frequency was defined based on the current prevalence of PAH of 15–50 cases/million individuals and accounting for reduced penetrance [1, 2].

Similar to PM2, we considered the highest allele frequency among ancestral subgroups on gnomAD control datasets to apply BA1 as a stand-alone criterion for benign variants with an allele frequency > 1% or ≥ 0.1% for BS1, an allele frequency which was greater than expected for PAH in controls. The presence of homozygotes was scored with BS2 as supporting for ≥ 2 controls and as strong for ≥ 3 controls.

#### 3.1.2. Relevant Variant Frequency in Patients: PS4, PP1, and BS4

Various criteria address the variant frequency in related or unrelated H/IPAH patients. While disease-causing *BMPR2* variants are often novel, a recurrence in unrelated individuals can be used as evidence for criterion PS4. If a variant has a very low population frequency (PM2, see above) and the same variant has been identified previously in > 4 unrelated H/IPAH patients, PS4_strong can be applied (Table 1). The criterion is moderate for > 3 previously described cases and supporting for > 1. Recurrent variants were identified through ClinVar, together with unpublished data from the labs of VCEP members and a thorough literature search for published variants not deposited in ClinVar. Importantly, care was taken to avoid counting duplicate reports of the same or related individuals across different publications. Also, cosegregation with disease lends strength to a variant. Analogous to the cardiomyopathy working group [17], we used PP1 as strong with ≥ 7 meioses, moderate for ≥ 5 meioses across or within families, and supporting for ≥ 3 meioses within a single family. In contrast, the lack of segregation of a variant with the disease in an affected PAH patient can be used as benign evidence for BS4. Due to the reduced penetrance of *BMPR2* variants, their presence in unaffected family members was not considered evidence for benignity.

#### 3.1.3. Null Variant Decision Tree: PVS1

The criterion with the strongest evidence for pathogenicity is PVS1 (Table 1). The general PVS1 decision tree [18] was adapted for *BMPR2* (see Supporting Information 1: Figure S1). The criterion applies to predicted null variants leading to a loss of function. Thus, it mainly refers to nonsense variants introducing a premature stop codon or frameshift variants and canonical splice site variants (+1, +2, −1, and −2) likely inducing nonsense-mediated decay (NMD). For such variants affecting *BMPR2* Exons 1 to 11 and all but the final 50 nucleotides of penultimate Exon 12 (c.1_c.2816), “PVS1_very strong” could be applied. A scoring as “strong” was possible if splice site variants were located in the canonical splice donor or acceptor site (positions +1, +2, −1, or −2) and were predicted to result in exon skipping, leading to an in-frame transcript with a loss or truncation of the ligand domain p.33_131, the transmembrane domain p.151_171, the kinase domain p.203_504, or the heterodimerization domain p.485_492.

#### 3.1.4. Functional Assay Characterisation: PS3 and BS3

The PS3 criterion involves in vitro or in vivo functional studies supportive of a damaging effect on the *BMPR2* gene or protein according to the ACMG guidelines. To determine whether a variant met the PS3 criterion, we considered quantitative and qualitative assays, as described below. To be considered valid evidence, quantitative assays needed to include biological and technical replicates, positive controls (wild-type *BMPR2*/BMPR2 and endogenous or total protein), validation controls (previously established, known pathogenic or benign variants within the same assay), negative controls (empty vectors), and robust statistical analysis (usually *t*-test or ANOVA). Assessment of functional effects did not need to be binary; statistically supported partial effects were also considered. Qualitative cytoplasmic retention assays did not require statistical analysis to apply PS3 as long as they included appropriate controls and replicates. Functional studies that failed to include validation controls could apply PS3 at the supporting level [19] at the discretion of the curator and PH VCEP.

##### 3.1.4.1. Gene Reporter/Luciferase Assays

Luciferase assays may be employed to investigate the functional impact of missense variants. Typically, cells are transfected with a combination of plasmids containing (i) BMP-responsive element from a SMAD promoter incorporated with a luciferase gene; (ii) wild-type and mutant BMPR2, for example, introduced by site-directed mutagenesis; (iii) and cognate Type I receptors (ALK1/2/3/6). Luciferase activity is then measured and normalised to beta-galactosidase or alkaline phosphatase activity. When compared to wild-type BMPR2, these analyses either confirmed abrogation of SMAD-mediated signalling activity for previously established kinase-dead variants or identified variants that retained enzymatic activity with a luciferase signal comparable to wild type [20, 21].

##### 3.1.4.2. Cell Proliferation Assays

The gold standard for assessing changes in cell proliferation is to quantify the percentage of DNA-synthesizing cells. This can be accomplished by measuring the incorporation of thymidine, or thymidine analogs bromodeoxyuridine (BrdU) or 5-ethynyl-2⁣′-deoxyuridine (EdU), into de novo DNA. In PAH, the proliferation of human pulmonary arterial smooth muscle, endothelial, or microvascular endothelial cells from H/IPAH transplant patients is compared to unaffected controls (i.e., unused donor tissue). The proliferation of similar cell types from mice heterozygous for patient-specific *Bmpr2* variants can be compared to wild type [22]. Growth curve proliferation assays involving direct cell counting are often included as an independent assessment of cell proliferation [22, 23]. Basic controls should include full growth medium with 10% fetal bovine serum (FBS) (positive control) and serum-restricted medium with 0.1% FBS (negative).

##### 3.1.4.3. Protein Binding Assays

Protein binding assays are designed to investigate the interaction of a protein with another protein, ligand–receptor binding, or transient protein binding for signal transduction. The acceptable protein assays in our PH VCEP panel were qualitative immunoprecipitation, glutathione S-transferase (GST) pull-down, and quantitative radioligand binding assays [20, 21, 24–28]. We considered confirmed ligands (bone morphogenetic proteins, BMPs) or coreceptors (activin receptors, ALKs) as acceptable binding partners, while unconfirmed ligands or receptors were not scored. Variant location within BMPR2 domains, for example, ligand binding domain or kinase domain, was also taken into consideration during the assessment of protein binding assays [21, 26, 28]. In addition, downstream effector readouts were limited to SMADs and p38, while any unconfirmed molecules were not assessed. Positive control of wild-type BMPR2 and negative control of empty vector were considered for upgrading or downgrading the functional criteria.

##### 3.1.4.4. SMAD Phosphorylation Assays

For SMAD phosphorylation assays, the ability of a BMPR2 variant to phosphorylate SMAD proteins should be compared to controls, preferably unphosphorylated total SMAD proteins or alternatively, housekeeping genes on a western blot with densitometry. Negative controls were either cells without transfected BMPR2, with BMPR2 wild type, or cells from non-PAH controls. Variants that demonstrated no deleterious effect with levels comparable to wild-type BMPR2 were scored under BS3 or BS3_supporting if validation controls were lacking.

#### 3.1.5. Definition of Critical Domains: PM1

For the PM1 criterion, critical BMPR2 functional domains were defined as the extracellular or ligand binding domain and serine–threonine kinase domain, containing a heterodimerization motif, separated by a transmembrane domain. These domains are delineated by strong evidence from structural studies of the activin Type II receptor extracellular domain, evolutionary conservation across eukaryotic kinases, and hydrogen deuterium exchange mass spectrometry analysis of the interface between BMPR2 and the ALK2 kinase domain. Moreover, gene reporter assays have indicated the relative importance of amino acid residues within these regions [20, 21, 29–31]. Ten cysteine residues within the extracellular domain are required for formation of five disulphide bonds essential for correct three-dimensional folding (p.Cys34, p.Cys60, p.Cys66, p.Cys84, p.Cys94, p.Cys99, p.Cys116, p.Cys117, p.Cys118, and p.Cys123). Sequences from 60 aligned eukaryotic kinases indicate the presence of eight evolutionarily invariant residues (p.Gly212, p.Lys230, p.Asp333, p.Asn338, p.Asp351, p.Glu386, p.Asp405, and p.Arg491) and four nearly invariant residues (p.Gly210, p.Glu/Asn245, p.Gly353, and p.Gly410). Moreover, structural analyses demonstrated that BMPR2/ALK heterodimerization is contingent on amino acids p.Asp485_Leu492 [29–31]. Variants located at these critical amino acid residues were upgraded to PM1_strong. The transmembrane domain was considered a critical domain for loss-of-function variants (nonsense/frameshift/splice site) involving Exon 4 but not for missense variants.

#### 3.1.6. Minor Adaptations: PM5 and PS1

In this study, PM5 at the moderate level was applied when a missense variant was found in the same amino acid as a previously described pathogenic missense variant. If the previous missense variant was classified as Likely Pathogenic, then PM5 was applied at the supporting level. PS1 could be applied as strong if the same amino acid substitution was described previously as pathogenic and as moderate if the variant was considered Likely Pathogenic. Alternative variants were curated using our PH VCEP specifications rather than relying on existing interpretations published in ClinVar or elsewhere. Similarly, PS1 may be applied to exonic or intronic variants with predicted splicing effects based on similarity with a previous pathogenic or Likely Pathogenic variant, weighted according to the ClinGen SVI Splicing Subgroup recommendations [32].

#### 3.1.7. In Silico Tools: PP3/BP4/BP7

The PP3 and BP4 criteria consider the computational evidence that supports the presence (PP3) or absence (BP4) of a predicted, deleterious effect on *BMPR2*. Our VCEP adopted the rare exome variant ensemble learner (REVEL) [33], SpliceAI [34], and combined annotation-dependent depletion (CADD) [35] scores to apply PP3 (REVEL ≥ 0.75; SpliceAI ≥ 0.2) or BP4 (REVEL ≤ 0.25; SpliceAI ≤ 0.1). In cases when REVEL or SpliceAI scores were not available, a CADD ≥ 20 (PP3) or ≤ 10 (BP4) was considered [35, 36]. SpliceAI ≤ 0.1 was used to apply BP7 for both synonymous and deep intronic variants and could be used together with BP4, as recommended by the ClinGen SVI Working Group.

#### 3.1.8. Other Criteria and Overall Scoring

Specifications for all criteria are detailed in Table 1 and Supporting Information 1: Figure S1. The de novo criteria (PS2 and PM6) and deletion/insertion change criteria (PM4 and BP3) were applied unaltered. PM3, PP2, PP4, BP1, BP2, and BP5 were considered not applicable to *BMPR2*-related PAH. Criteria referring to a “reputable source” (PP5 and BP6) are no longer used according to ClinGen revised guidance [37]. Criteria were combined using the Bayesian points scoring system outlined by Tavtigian et al. [38] with a modification for likely benign (LB), as follows: Pathogenic (P), ≥ 10 points; Likely Pathogenic, 6 to 9; variant of uncertain significance (VUS), −1 to 5; Likely Benign, −2 to −6; and Benign (B), ≤ −7.

### 3.2. Pilot Testing of PH VCEP Specifications

**Figure 2:**
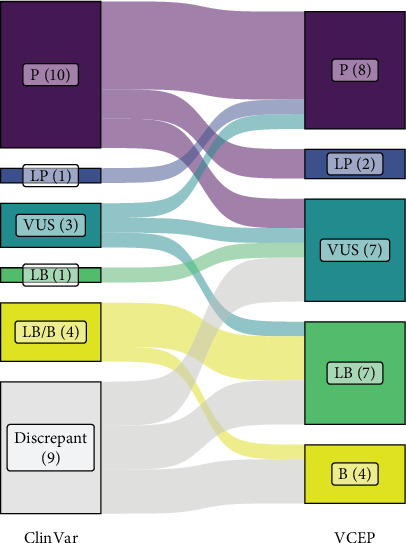
Comparison of ClinVar and PH VCEP classifications. Sankey plot shows the effect of applying our VCEP variant specifications (right) compared with the original ClinVar classification(s) (left) for the pilot panel of 28 *BMPR2* variants. P, pathogenic; LP, likely pathogenic; VUS, variant of uncertain significance; LB, likely benign; B, benign.

The results of our pilot curation of 28 *BMPR2* variants are detailed in Table 2 and Figure 2. The classification of seven variants (25%; six pathogenic and one VUS) did not change from the existing ClinVar classification. These included well-established Pathogenic variants that were included as “positive controls” to test our specifications. If the P/LP and B/LB classifications are grouped per their clinical utility, a further seven variants were unchanged, including four with mixed B/LB classifications in ClinVar. Three of these were resolved to Likely Benign and one to Benign when our specifications were applied, although this is not consequential for their clinical interpretation. Of the remaining 14 variants (50%), nine had discrepant classifications in ClinVar. Eight of these were VUS/LB or VUS/LB/B, of which three were resolved to Benign, three to Likely Benign, and two remained as VUS. Two variants changed from Likely Benign to VUS or vice versa. The most pronounced and clinically consequential changes were seen with intronic splice region variants, with three variants (c.247+1_247+7del, c.529+2T>C, and c.968-3C>G) reclassified from pathogenic or P/VUS to VUS and one from VUS to Pathogenic (c.968-5A>G). As discussed further below, mRNA analysis to confirm the predicted consequences of splice site variants is critical for accurate classification, especially for variants outside of the canonical positions.

### 3.3. Evaluation of Newer Bioinformatics Tools: AlphaMissense and BayesDel

Lastly, we performed a pilot evaluation of two newer bioinformatic prediction tools not covered in the original ACMG/AMP guidelines. As a novel and emerging in silico prediction tool for missense variants, AlphaMissense, developed by Google DeepMind, calculates missense variant pathogenicity by combining structural context and evolutionary conservation, using data from AlphaFold prediction and human and primate variant frequency databases [39]. It generates scores between 0 and 1, with higher scores predicting a greater likelihood of pathogenicity. Suggested thresholds for Likely Pathogenic and Likely Benign are > 0.564 and < 0.340, respectively. BayesDel (no AF) is a deleteriousness metascore. The range of the score is from −1.29334 to 0.75731. The higher the score, the more likely the variant is pathogenic [40]. Both programs were applied, in addition to REVEL and CADD, to the 15 missense variants in our pilot trial and compared to our final VCEP classification. In this preliminary analysis, AlphaMissense outperformed REVEL, CADD, and BayesDel by reaching an agreement with our experts for 12/15 variants, while the other three programs each only supported 5/15 assessments (Table 3).

## 4. Discussion

We adapted the general ACMG/AMP variant specification criteria specifically to *BMPR2*, the gene most commonly implicated in PAH, with loss or reduced function as the mechanism of action. This guidance should be valuable to improve harmonization in reporting variants identified in patients with heritable or idiopathic PAH across clinical laboratories. Of note, we did not consider variants identified in patients with associated PAH; therefore, these specifications may require modification in such cases.

Unlike null variants such as canonical splice site or frameshift variants, it is often challenging to classify novel and rare missense variants as Likely Pathogenic/Pathogenic with currently available data. In many cases, functional data are lacking, and previous reports of the same variant in other PAH patients are usually limited. This often leaves only PM2 (low population frequency) and PP3 (pathogenicity predictions) to support missense variant interpretation. Hence, it is crucial for patients and clinicians that all variants are uploaded to ClinVar to potentially identify recurrent variants or amino acid changes to apply additional criteria (PS4, PS1, and PM5). Accurate predictive testing for the familial disease-causing variant is only possible for Likely Pathogenic or Pathogenic variants, and genetic risk prediction is critical as there are increasingly effective therapies for PAH that are likely more effective when applied earlier in the disease course.

Of the 28 *BMPR2* variants curated in this pilot study, only eight (28.6%) had functional analyses that could be scored, of which five provided evidence for PS3, two for BS3, and one for BS3_supp. Access to tissues, cell lines, and nucleic acids to perform the functional analyses considered in this study is not always possible, and, when available, the materials and resources may be limited. The lack of functional analyses presents challenges when determining the impact of a given reported genomic variant. In this regard, the assessment of mRNA in patients with suspected splice site variants located more than two base pairs within the intron is relatively straightforward and provides a key experimental indication of pathogenicity. The importance of this is highlighted by three splice site variants in our pilot panel, including one at the +2 position of a splice donor site, that were downgraded from Pathogenic to VUS due to the lack of supporting mRNA data. Conversely, one variant in the −5 splice acceptor position was upgraded from VUS to Pathogenic on the basis of mRNA analysis, performed in the lab of a VCEP member, that provided evidence for PVS1 (RNA).

Our pilot testing of AI-based prediction algorithms, including AlphaMissense, suggests that such programs may be highly accurate in predicting the pathogenicity of missense variants in the absence of functional data. However, this is an evolving field, with ongoing development of new programs and calibration of threshold values for established tools. Of note, while this manuscript was in revision, a new posterior probability-based calibration was proposed for AlphaMissense [41]. The minimal threshold to apply PP3 was determined as ≥ 0.792, considerably higher than the original 0.564, while the threshold for BP4 moved from 0.340 to < 0.170. As a result, the agreement with our VCEP classification for *BMPR2* missense variants was reduced to 7 of 15 variants, with five previously concordant variants changing from Likely Benign to uncertain (Supporting Information 2: Table S1). Nonetheless, Bergquist et al. emphasize that these tools should not be considered the sole classifier; rather, an integrated analysis per the ACMG framework will almost always be needed [41]. With this in mind, we reframed our comparison of these algorithms with our integrated VCEP curation to focus on whether the score produced by each program was concordant, neutral, or discordant with the final VCEP classification, rather than applying a definitive in silico classification. Uncertain calls were considered neutral. The updated thresholds rendered AlphaMissense more conservative in calling evidence for benignity (BP4), with only 2/8 Benign/Likely Benign variants achieving BP4 and the remaining six having indeterminate scores. Importantly, all Pathogenic/Likely Pathogenic variants still met the minimal threshold for PP3, and no AlphaMissense calls were discordant (Supporting Information 2: Table S1). In contrast, CADD scores, even when recalibrated to the more stringent thresholds proposed by Pejaver et al. [40], were discordant for five variants, while the recalibrated thresholds for REVEL and BayesDel [41] were discordant in three and five cases, respectively. While these results suggest that AlphaMissense and REVEL are promising tools, the use of in silico prediction programs is a dynamic landscape that will require ongoing reevaluation. Therefore, the composite approach adopted in our *BMPR2* guidelines remains the most robust method of determining variant pathogenicity or benignity [40, 41].

In this study, we encountered a large Iberian family in which 32 individuals were genotyped and 22 were heterozygous for c.1472G>A p.Arg491Gln cosegregating with PAH [42]. However, segregation analyses are typically challenging due to small family sizes, the work needed to organize family studies, and incomplete penetrance. Due to the need for cardiac catheterization for clinical diagnosis, pulmonary hemodynamics may not be available for some family members. In addition, without enough affected heterozygotes, the number of meioses observed may not be sufficient to use the segregation criteria PP1 or BS4. The presence or absence of this evidence can significantly influence the final classification of a given variant. Therefore, it is essential to develop protocols or policies to collect relevant data from large families when there is suspicion of HPAH. Moreover, clinical guidelines recommend offering cascade testing to potentially identify at-risk heterozygotes and offer clinical screening to diagnose PAH as early as possible [5].

ACMG guidelines were developed for Mendelian genetic disorders with the goal of dichotomizing variants into Pathogenic/Likely Pathogenic and Benign/Likely Benign categories to define their clinical utility (or lack thereof). Most heritable PAH cases, including those related to *BMPR2*, show autosomal dominant inheritance, and thus, our VCEP appropriately sought to adapt ACMG guidelines for this gene. However, PAH shows reduced penetrance, suggesting that other genetic and/or environmental modifiers also play a role. In this regard, the publication by Masson et al. is thought provoking, recognizing that some VUS may demonstrate disease relevance based on population genetics or functional data but do not satisfy the stringent criteria for Pathogenic or Likely Pathogenic classification [43]. Thus, they proposed new terminology of “predisposing” and “likely predisposing.” This concept may be helpful in classifying lower penetrance variants that could contribute to PAH in a polygenic model and should be considered in the future.

In conclusion, these newly adapted ClinGen specification guidelines to assess *BMPR2* variants for PAH patients will allow more reliable and accurate variant classification. Although the overall proportion of VUS increased, this reflects that approximately one-third of the chosen variants had discrepant classifications in ClinVar. Notably, we were able to resolve six of the eight variants with a discrepant VUS/LB, reclassifying them as Benign or Likely Benign. The remaining VUS may be reclassified with increasing evidence in the future. Benign or Likely Benign variants are most likely unrelated to the disease and thus do not increase the genetic risk of developing PAH. In contrast, Pathogenic or Likely Pathogenic variants allow for risk stratification of other family members. Thus, the consensus specification document substantially aids in providing patients and caregivers with an accurate genetic diagnosis which may play an increasing role in therapy.

## Figures and Tables

**Table 1:** Adapted *BMPR2* variant specifications for pulmonary arterial hypertension.

**Pathogenic criteria**		
**Criteria**	**Criteria description**	**Specification**

Very strong criteria		
PVS1	Null variant in a gene where loss of function is a known mechanism of disease	Use *BMPR2*-specific PVS1 decision tree (Supporting Information 1: Figure S1)

Strong criteria		
PS1	Same amino acid change as a previously established Pathogenic variant regardless of nucleotide change	Downgrade to PS1_mod if variant is likely pathogenic
PS2	De novo (paternity confirmed) in a patient with the disease and no family history	—
PS3	Well-established in vitro or in vivo functional studies supportive of a damaging effect	If no validation controls (known pathogenic or benign variants), downgrade to PS3_supp If the same functional assay has been performed for the same variant by an independent group and demonstrated to have the same functional effect by both groups, then PS3_strong Can be applied additively with PP3
PS4	The prevalence of the variant in affected individuals is significantly increased compared with the prevalence in controls	> 4 probands: PS4_strong > 3 probands: PS4_mod > 1 proband PS4_supp PM2 supporting must be met. Affected individual = mPAP >20 mmHg by the right heart catheterization or estimated by echocardiography if RHC is not advised. PS4 (at any strength) and PM2_supp can be used additively

Moderate criteria		
PM1	Located in a mutational hot spot and/or critical and well-established functional domain	Extracellular domain: p.Cys34, Cys60, Cys66, Cys84, Cys94, Cys99, Cys116, Cys117, Cys118, and Cys123. Kinase domain: p.Gly210, Gly212, Lys230, Glu/Asn245, Asp333, Asn338, Asp351, Gly353, Glu386, Asp405, Gly410, and Arg491. Heterodimerization: p.Asp485, Gln486, Asp487, Ala488, Arg489, Ala490, Arg491, and Leu492
PM2	Absent/rare from controls in an ethnically matched cohort population sample Note: We are deviating from “ethnically matched cohort” because demographic/genetic ancestry data are not always available from ClinVar and other sources	< 0.01% among gnomAD controls, using the subpopulation with the highest frequency and at least 1000 allele counts. Always apply as PM2_supp PM2_supp and PS4 (at any strength) can be used additively
PM3	For recessive disorders, detected in trans with a pathogenic variant	N/A
PM4	Protein length changes due to in-frame deletions/insertions in a nonrepeat region or stop-loss variants	—
PM5	Missense change at an amino acid residue where a different missense change determined to be pathogenic has been seen before	If the second variant is pathogenic, then apply PM5_mod. If the second variant is likely pathogenic, then apply PM5_supp
PM6	Confirmed de novo without confirmation of paternity and maternity	—

Supporting criteria		
PP1	Cosegregation with disease in multiple affected family members Note: At least three meioses/family required	≥ 7 meioses: PP1_strong ≥ 5 meioses: PP1_mod ≥ 3 meioses in a single family: PP1_supp
PP2	Missense variant in a gene that has a low rate of benign missense variation and where missense variants are a common mechanism of disease	N/A
PP3	Multiple lines of computational evidence support a deleterious effect on the gene or gene product	REVEL ≥ 0.75 for missense, no up/downgrading; SpliceAI ≥ 0.2 for noncanonical splice variants. If no REVEL score or SpliceAI prediction available, then CADD ≥ 20 can be used. PP3 can be applied additively with PS3
PP4	Phenotype specific for disease with single genetic etiology	N/A
PP5	Reputable source recently reports variant as pathogenic, but the evidence is not available to the laboratory to perform an independent evaluation	N/A not to be used

**Benign criteria**		
**Criteria**	**Criteria description**	**Specification**

Stand-alone criteria		
BA1	Allele frequency above X%	1%

Strong criteria		
BS1	Allele frequency greater than expected for disease (> X%) Note: PAH prevalence: 15–50 cases/million individuals	≥ 0.1% among gnomAD controls, using the subpopulation with the highest frequency and at least 1000 allele counts
BS2	Observed in the homozygous state in a healthy adult	≥ 3 counts: BS2_strong; ≥ 2 counts: BS2_supp
BS3	Well-established in vitro or in vivo functional studies shows no damaging effect on protein function	BMPR2 functional assay documentation with appropriate controls
BS4	Lack of segregation in affected members of a family	Lack of disease segregation among variant heterozygotes should not be scored due to low penetrance of PAH variants in families. Lack of variant segregation in affected family members should be scored

Supporting criteria		
BP1	Missense variant in gene where only loss-of-function causes disease	N/A
BP2	Observed in trans with a pathogenic variant for a fully penetrant dominant gene/disorder; or observed in cis with a pathogenic variant in any inheritance pattern	N/A
BP3	In-frame deletions/insertions in a repetitive region without a known function	—
BP4	Multiple lines of computational evidence suggest no impact on gene or gene product	REVEL ≤ 0.25, no up/downgrading. SpliceAI ≤ 0.1 for noncanonical splice variants. If no REVEL score or SpliceAI prediction is available, then CADD ≤ 10 can be used
BP5	Variant found in a case with an alternate molecular basis for disease	N/A
BP6	Reputable source recently reports variant as benign, but the evidence is not available to the laboratory to perform an independent evaluation	N/A not to be used
BP7	A synonymous (silent) variant for which splicing prediction algorithms predict no impact to the splice consensus sequence nor the creation of a new splice site and the nucleotide is not highly conserved	—

Abbreviations: mod, moderate; PAH, pulmonary arterial hypertension; supp, supporting; VUS, variant of uncertain significance.

**Table 2:** Detail of the *BMPR2* variants curated in the pilot study.

**Variant** ^**a**^	**Variant type**	**ClinVar classification**	**PH VCEP classification**	**ACMG/AMP criteria and points applied**	**Score** ^**b**^
c.-924A>G	5⁣′-Untranslated region	VUS/likely benign	Benign	BA1 −8, BS2_supp −1, BP4 −1	−10
c.-669G>A	5⁣′-Untranslated region	Likely benign/benign	Benign	BA1 −8	−8
c.218C>G (p.Ser73⁣^∗^)	Nonsense	Pathogenic	Pathogenic	PVS1 +8, PS4_supp +1, PM2_supp +1	+10
c.240_241insT (p.Lys81⁣^∗^)	Nonsense	Pathogenic	Likely Pathogenic	PVS1 +8, PM2_supp +1	+9
c.247+1_247+7del	Splice region/intronic	Pathogenic	VUS	PVS1_strong +4, PM2_supp +1	+5
c.251G>A (p.Cys84Tyr)	Missense	Pathogenic	Pathogenic	PS4_mod +2, PM1_strong +4, PM2_supp +1, PM5 +2, PP3 +1	+10
c.354T>G (p.Cys118Trp)	Missense	Pathogenic (3)	Pathogenic	PS3 +4, PS4 +4, PM1_strong +4, PM2_supp +1, PM5_supp +1, PP1 +1, PP3 +1	+16
c.419-38del	Splice region/intronic	VUS/likely benign/benign	Benign	BA1 −8, BS2 −4, BP4 −1, BP7 −1	−14
c.529+2T>C	Canonical splice site	Pathogenic	VUS	PVS1_strong +4, PM2_supp +1	+5
c.545G>A (p.Gly182Asp)	Missense	VUS (3)/likely benign (1)	Likely benign	BS3 −4, PP3 +1	−3
c.797G>C (p.Arg266Thr)	Missense	VUS	VUS	PM2_supp +1	+1
c.901T>C (p.Ser301Pro)	Missense	Likely pathogenic	Pathogenic	PS3 +4, PS4 +4, PM1 +2, PM2_supp +1, PM6 +2	+13
c.968-5A>G	Splice site/intronic	VUS (3)	Pathogenic	PVS1 (RNA) +8, PS4_supp +1, PM2_supp +1	+10
c.968-3C>G	Splice site/intronic	Pathogenic/VUS	VUS	PS4_supp +1, PM2_supp +1, PP3 +1	+3
c.1040G>A (p.Cys347Tyr)	Missense	Pathogenic	Likely pathogenic	PS3_supp +1, PS4 +4, PM2_supp +1, PM5_supp +1, PP3 +1	+8
c.1042G>A (p.Val348Ile)	Missense	Likely benign/benign	Likely benign	BS1 −4, PS3_supp +1	−3
c.1413+1G>A	Canonical splice site	Pathogenic	Pathogenic	PVS1 +8, PS4_mod +2, PM2_supp +1	+11
c.1424C>A (p.Ser475⁣^∗^)	Nonsense	Pathogenic (2)	Pathogenic	PVS1 +8, PS4_supp +1, PM2_supp +1	+10
c.1472G>A (p.Arg491Gln)	Missense	Pathogenic	Pathogenic	PS2 +4, PS3_supp +1, PS4 +4, PM1_strong +4, PM2_supp +1, PM5 +2, PP1 +1, PP3 +1	+18
c.1481C>T (p.Ala494Val)	Missense	Likely benign	VUS	PM1 +2, PP3 +1, BS1 −4	−1
c.1509A>C (p.Glu503Asp)	Missense	VUS	Likely benign	BS3 −4	−4
c.1698T>A (p.Ile566=)	Synonymous	VUS/likely benign	VUS	PM2_supp +1, BP4 −1, BP7 −1	−1
c.1766A>G (p.Tyr589Cys)	Missense	Likely benign/benign	Likely benign	BS1 −4	−4
c.2186G>C (p.Gly729Ala)	Missense	VUS/likely benign	Likely benign	BS1 −4	−4
c.2352C>T (p.Val784=)	Synonymous	VUS/likely benign	VUS	PM2_supp +1, BP4 −1, BP7 −1	−1
c.2618G>A (p.Arg873Gln)	Missense	VUS/likely benign	Benign	BS1 −4, BS3_supp −1, BS4 −4	−9
c.2887G>T (p.Gly963Cys)	Missense	Likely benign/benign	Likely benign	BS1 −4	−4
c.2948G>A (p.Arg983Gln)	Missense	VUS (3)/likely benign (1)/benign (1)	Likely benign	BS1 −4	−4

Abbreviations: mod, moderate; supp, supporting; VUS, variant of uncertain significance.

^a^Variant nomenclature refers to transcript NM_001204.7.

^b^Scoring modified from Tavtigian et al. [38]: pathogenic (P), ≥ 10 points; likely pathogenic (LP), 6 to 9; VUS, −1 to 5; likely benign (LB), −2 to −6; benign (B), ≤ −7.

**Table 3:** Comparison of three in silico prediction programs with our VCEP classification of missense variants.

**Variant** ^**a**^	**PHVCEP classification**	**REVEL**	**CADD**	**AlphaMissense**	**BayesDel**
c.251G>A (p.Cys84Tyr)	Pathogenic	Deleterious (0.95)	Deleterious (32)	Likely pathogenic (0.998)	Deleterious (strong) (0.59)
c.354T>G (p.Cys118Trp)	Pathogenic	Deleterious (0.93)	Deleterious (28.5)	Likely pathogenic(0.999)	Deleterious (strong) (0.53)
c.545G>A (p.Gly182Asp)	Likely benign	Deleterious (0.81)	Deleterious (28.0)	Uncertain (0.425)	Deleterious (moderate) (0.36)
c.797G>C (p.Arg266Thr)	VUS	Uncertain (0.63)	Deleterious (29.5)	Likely pathogenic(0.893)	Deleterious (supporting) (0.21)
c.901T>C (p.Ser301Pro)	Pathogenic	Uncertain (0.45)	Deleterious (25.1)	Likely pathogenic(0.905)	Deleterious (supporting) (0.16)
c.1040G>A (p.Cys347Tyr)	Likely pathogenic	Deleterious (0.94)	Deleterious (31)	Likely pathogenic (0.994)	Deleterious (moderate) (0.48)
c.1042G>A (p.Val348Ile)	Likely benign	Uncertain (0.74)	Deleterious (26.2)	Likely benign(0.201)	Deleterious (moderate) (0.39)
c.1472G>A (p.Arg491Gln)	Pathogenic	Deleterious (0.96)	Deleterious (34)	Likely pathogenic(0.992)	Deleterious (strong) (0.62)
c.1481C>T (p.Ala494Val)	VUS	Deleterious (0.87)	Deleterious (31)	Likely pathogenic(0.979)	Deleterious (moderate) (0.4)
c.1509A>C (p.Glu503Asp)	Likely benign	Uncertain (0.66)	Uncertain (17.7)	Likely benign (0.26)	Deleterious (moderate) (0.29)
c.1766A>G (p.Tyr589Cys)	Likely benign	Uncertain (0.58)	Deleterious (28)	Likely benign (0.263)	Deleterious (supporting) (0.18)
c.2186G>C (p.Gly729Ala)	Likely benign	Uncertain (0.36)	Deleterious (23.6)	Likely benign (0.101)	Uncertain (0.06)
c.2618G>A (p.Arg873Gln)	Benign	Uncertain (0.55)	Deleterious (26)	Likely benign (0.266)	Deleterious (moderate) (0.34)
c.2887G>T (p.Gly963Cys)	Likely benign	Uncertain (0.42)	Deleterious (26.8)	Likely benign (0.099)	Uncertain (−0.04)
c.2948G>A (p.Arg983Gln)	Likely benign	Uncertain (0.4)	Deleterious (24.8)	Likely benign (0.321)	Uncertain (−0.04)

**Agreement with VCEP**	—	5/15	5/15	12/15	5/15

*Note:* AlphaMissense: likely pathogenic, 0.564–1.0; uncertain, 0.34–0.564; and likely benign, 0–0.34. BayesDel: ≥ 0.5, strong pathogenic evidence; 0.27–0.5, moderate pathogenic; 0.13–0.27, supporting pathogenic; −0.36 to −0.18, supporting benign; and score ≤ −0.36, moderate benign [40]. CADD: ≥ 20 pathogenic, 10–20 uncertain, and ≤ 10 benign. REVEL: ≥ 0.75 pathogenic, 0.25–0.75 uncertain, and ≤ 0.25 benign.

Abbreviation: VUS, variant of uncertain significance.

^a^Variant nomenclature refers to transcript NM_001204.7.

## Data Availability

The *BMPR2* specifications are available in the ClinGen Criteria Specification Registry (https://cspec.genome.network/cspec/ui/svi/doc/GN125). Variant curations were published to the ClinGen Evidence Repository and submitted to ClinVar (https://www.ncbi.nlm.nih.gov/clinvar/?term=bmpr2%255Bgene%255D%26redir=gene).

## Supporting Information

Supporting Information 1 Figure S1: PVS1 decision tree adapted for *BMPR2.*

Supporting Information 2 Table S1: Revised comparison of three in silico prediction programs with our VCEP classification using updated calibration thresholds.
