# Supplementary material for: Adaptation of ACMG/AMP Guidelines for Clinical Classification of BMPR2 Variants in Pulmonary Arterial Hypertension Resolves Variants of Unclear Pathogenicity in ClinVar
Source: Hum Mutat. 2025 Jul 6;2025:2475635. doi: 10.1155/humu/2475635 (PMC12256177; doi:10.1155/humu/2475635)
Supplement: Supporting Information 1 — Figure S1: PVS1 decision tree adapted for BMPR2. [file 2475635.f1.pptx]

## Slide 1
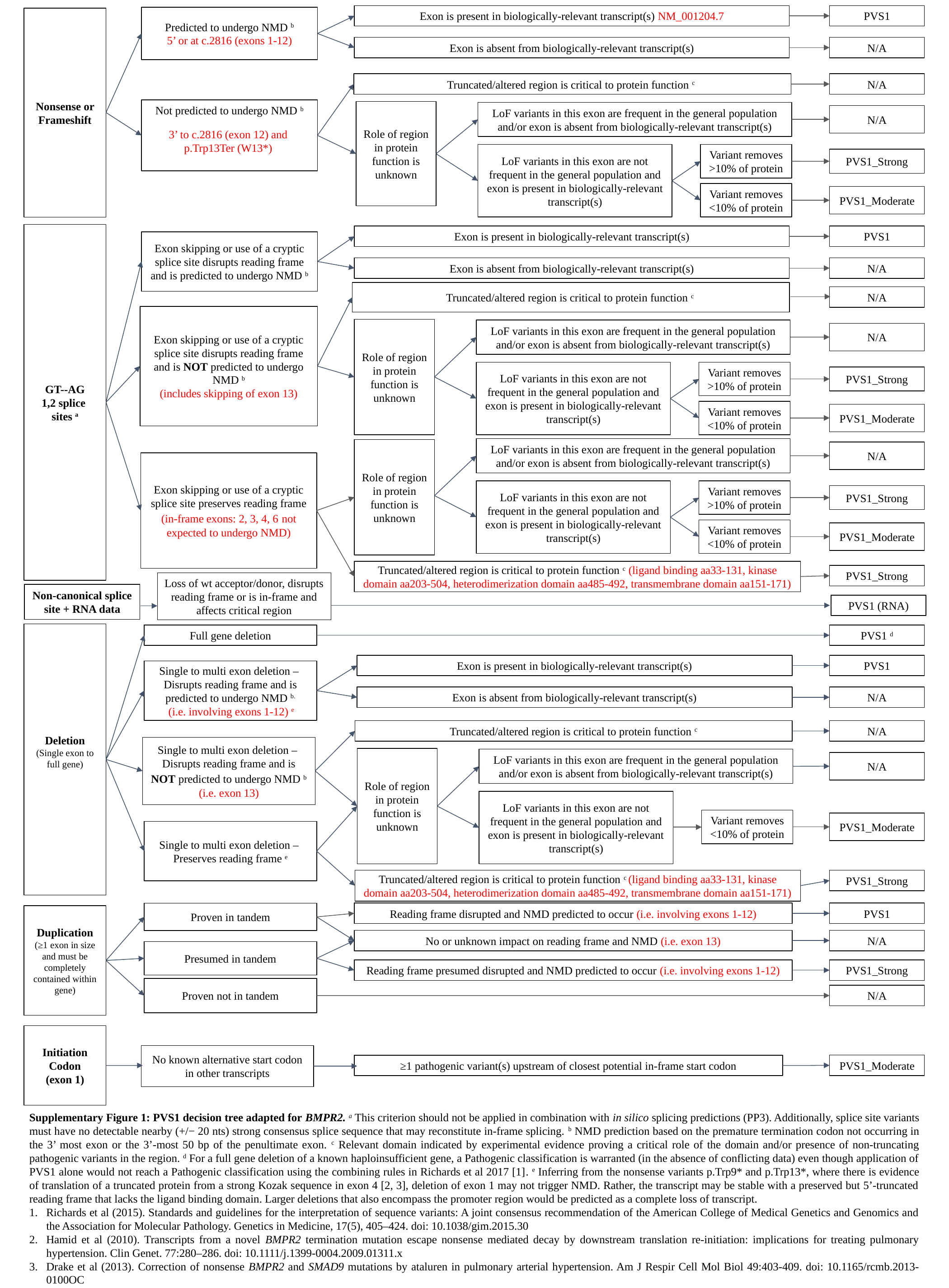

Exon is present in biologically-relevant transcript(s) NM_001204.7
PVS1
Predicted to undergo NMD b
5’ or at c.2816 (exons 1-12)
Nonsense or Frameshift
Exon is absent from biologically-relevant transcript(s)
N/A
Truncated/altered region is critical to protein function c
N/A
Not predicted to undergo NMD b
Role of region in protein function is unknown
LoF variants in this exon are frequent in the general population and/or exon is absent from biologically-relevant transcript(s)
N/A
3’ to c.2816 (exon 12) and p.Trp13Ter (W13*)
LoF variants in this exon are not frequent in the general population and exon is present in biologically-relevant transcript(s)
Variant removes >10% of protein
PVS1_Strong
Variant removes <10% of protein
PVS1_Moderate
GT--AG
1,2 splice
sites a
Exon is present in biologically-relevant transcript(s)
PVS1
Exon skipping or use of a cryptic splice site disrupts reading frame and is predicted to undergo NMD b
Exon is absent from biologically-relevant transcript(s)
N/A
Truncated/altered region is critical to protein function c
N/A
Exon skipping or use of a cryptic splice site disrupts reading frame and is NOT predicted to undergo NMD b
(includes skipping of exon 13)
Role of region in protein function is unknown
LoF variants in this exon are frequent in the general population and/or exon is absent from biologically-relevant transcript(s)
N/A
LoF variants in this exon are not frequent in the general population and exon is present in biologically-relevant transcript(s)
Variant removes >10% of protein
PVS1_Strong
Variant removes <10% of protein
PVS1_Moderate
LoF variants in this exon are frequent in the general population and/or exon is absent from biologically-relevant transcript(s)
Role of region in protein function is unknown
N/A
Exon skipping or use of a cryptic splice site preserves reading frame
(in-frame exons: 2, 3, 4, 6 not expected to undergo NMD)
LoF variants in this exon are not frequent in the general population and exon is present in biologically-relevant transcript(s)
Variant removes >10% of protein
PVS1_Strong
Variant removes <10% of protein
PVS1_Moderate
Truncated/altered region is critical to protein function c (ligand binding aa33-131, kinase domain aa203-504, heterodimerization domain aa485-492, transmembrane domain aa151-171)
PVS1_Strong
Loss of wt acceptor/donor, disrupts reading frame or is in-frame and affects critical region
Non-canonical splice site + RNA data
PVS1 (RNA)
Deletion
(Single exon to full gene)
Full gene deletion
PVS1 d
Exon is present in biologically-relevant transcript(s)
PVS1
Single to multi exon deletion –
Disrupts reading frame and is predicted to undergo NMD b.
 (i.e. involving exons 1-12) e
Exon is absent from biologically-relevant transcript(s)
N/A
Truncated/altered region is critical to protein function c
N/A
Single to multi exon deletion –
Disrupts reading frame and is NOT predicted to undergo NMD b (i.e. exon 13)
Role of region in protein function is unknown
LoF variants in this exon are frequent in the general population and/or exon is absent from biologically-relevant transcript(s)
N/A
LoF variants in this exon are not frequent in the general population and exon is present in biologically-relevant transcript(s)
Variant removes <10% of protein
PVS1_Moderate
Single to multi exon deletion –
Preserves reading frame e
Truncated/altered region is critical to protein function c (ligand binding aa33-131, kinase domain aa203-504, heterodimerization domain aa485-492, transmembrane domain aa151-171)
PVS1_Strong
Reading frame disrupted and NMD predicted to occur (i.e. involving exons 1-12)
PVS1
Proven in tandem
Duplication
(≥1 exon in size and must be completely contained within gene)
No or unknown impact on reading frame and NMD (i.e. exon 13)
N/A
Presumed in tandem
Reading frame presumed disrupted and NMD predicted to occur (i.e. involving exons 1-12)
PVS1_Strong
Proven not in tandem
N/A
Initiation Codon
(exon 1)
No known alternative start codon in other transcripts
PVS1_Moderate
≥1 pathogenic variant(s) upstream of closest potential in-frame start codon
Supplementary Figure 1: PVS1 decision tree adapted for BMPR2. a This criterion should not be applied in combination with in silico splicing predictions (PP3). Additionally, splice site variants must have no detectable nearby (+/− 20 nts) strong consensus splice sequence that may reconstitute in-frame splicing. b NMD prediction based on the premature termination codon not occurring in the 3’ most exon or the 3’-most 50 bp of the penultimate exon. c Relevant domain indicated by experimental evidence proving a critical role of the domain and/or presence of non-truncating pathogenic variants in the region. d For a full gene deletion of a known haploinsufficient gene, a Pathogenic classification is warranted (in the absence of conflicting data) even though application of PVS1 alone would not reach a Pathogenic classification using the combining rules in Richards et al 2017 [1]. e Inferring from the nonsense variants p.Trp9* and p.Trp13*, where there is evidence of translation of a truncated protein from a strong Kozak sequence in exon 4 [2, 3], deletion of exon 1 may not trigger NMD. Rather, the transcript may be stable with a preserved but 5’-truncated reading frame that lacks the ligand binding domain. Larger deletions that also encompass the promoter region would be predicted as a complete loss of transcript.
Richards et al (2015). Standards and guidelines for the interpretation of sequence variants: A joint consensus recommendation of the American College of Medical Genetics and Genomics and the Association for Molecular Pathology. Genetics in Medicine, 17(5), 405–424. doi: 10.1038/gim.2015.30
Hamid et al (2010). Transcripts from a novel BMPR2 termination mutation escape nonsense mediated decay by downstream translation re-initiation: implications for treating pulmonary hypertension. Clin Genet. 77:280–286. doi: 10.1111/j.1399-0004.2009.01311.x
Drake et al (2013). Correction of nonsense BMPR2 and SMAD9 mutations by ataluren in pulmonary arterial hypertension. Am J Respir Cell Mol Biol 49:403-409. doi: 10.1165/rcmb.2013-0100OC
