# Supplementary material for: Adaptation of ACMG/AMP Guidelines for Clinical Classification of BMPR2 Variants in Pulmonary Arterial Hypertension Resolves Variants of Unclear Pathogenicity in ClinVar
Source: Hum Mutat. 2025 Jul 6;2025:2475635. doi: 10.1155/humu/2475635 (PMC12256177; doi:10.1155/humu/2475635)
Supplement: Supporting Information 2 — Table S1: Revised comparison of three in silico prediction programs with our VCEP classification using updated calibration thresholds. [file 2475635.f2.pdf]

Supplementary Table 1: Revised comparison of three *in silico* prediction programmes with our VCEP classification using updated calibration thresholds

Variant*	PH VCEP classification	REVEL (our specifications)†	REVEL (Bergquist[1])	CADD (Pejaver[2])	AlphaMissense (Bergquist[1])	BayesDel (Bergquist[1])
c.251G>A (p.Cys84Tyr)	Pathogenic	PP3_supp (0.951)	PP3_strong	PP3_mod (32)	PP3_strong (0.998)	PP3_strong (0.59)
c.354T>G (p.Cys118Trp)	Pathogenic	PP3_supp (0.928)	PP3_mod	PP3_mod (28.5)	PP3_strong (0.999)	PP3_strong (0.53)
c.545G>A (p.Gly182Asp)	Likely Benign	PP3_supp (0.807)	PP3_mod ‡	PP3_supp (28.0)	Uncertain (0.425) ‡	PP3_mod (0.36)
c.797G>C (p.Arg266Thr)	VUS	Uncertain (0.628)	Uncertain	PP3_mod (29.5)	PP3_supp (0.893)	PP3_supp (0.21)
c.901T>C (p.Ser301Pro)	Pathogenic	Uncertain (0.451)	Uncertain	Uncertain (25.1)	PP3_supp (0.905)	PP3_supp (0.16)
c.1040G>A (p.Cys347Tyr)	Likely Pathogenic	PP3_supp (0.939)	PP3_strong	PP3_mod (31)	PP3_strong (0.994)	PP3_mod (0.48)
c.1042G>A (p.Val348Ile)	Likely Benign	Uncertain (0.744)	PP3_supp	PP3_supp (26.2)	Uncertain (0.201)	PP3_mod (0.39)
c.1472G>A (p.Arg491Gln)	Pathogenic	PP3_supp (0.962)	PP3_strong	PP3_mod (34)	PP3_strong (0.992)	PP3_strong (0.62)
c.1481C>T (p.Ala494Val)	VUS	PP3_supp (0.872)	PP3_mod	PP3_mod (31)	PP3_mod (0.979)	PP3_mod (0.4)
c.1509A>C (p.Glu503Asp)	Likely Benign	Uncertain (0.662)	PP3_supp	BP4_supp (17.7)	Uncertain (0.26)	PP3_mod (0.29)
c.1766A>G (p.Tyr589Cys)	Likely Benign	Uncertain (0.577)	Uncertain	PP3_supp (28)	Uncertain (0.263)	PP3_supp (0.18)
c.2186G>C (p.Gly729Ala)	Likely Benign	Uncertain (0.36)	Uncertain	Uncertain (23.6)	BP4_supp (0.101)	Uncertain (0.06)
c.2618G>A (p.Arg873Gln)	Benign	Uncertain (0.551)	Uncertain	PP3_supp (26)	Uncertain (0.266)	PP3_mod (0.34)
c.2887G>T (p.Gly963Cys)	Likely Benign	Uncertain (0.418)	Uncertain	PP3_supp (26.8)	BP4-mod (0.099)	Uncertain (-0.04)
c.2948G>A (p.Arg983Gln)	Likely Benign	Uncertain (0.4)	Uncertain	Uncertain (24.8)	Uncertain (0.321)	Uncertain (-0.04)
**Agreement with VCEP**	-	5/15	5/15	5/15	7/15	5/15
**Discordant with VCEP‡**		1/15	3/15	5/15	0/15	5/15

*Variant nomenclature refers to transcript NM_001204.7

†Our *BMPR2* guidelines did not allow up or downgrading, therefore all REVEL scores ≥0.75 were categorized as PP3_supp

‡Predictions that were discordant with the overall VCEP classification are shaded in pale grey. Uncertain predictions were considered neutral, since they do not influence the score in either direction.

[1]Thresholds and weights are from Bergquist et al. (2025). Calibration of additional computational tools expands ClinGen recommendation options for variant classification with PP3/BP4 criteria. Genetics in Medicine in press. doi: 10.1016/j.gim.2025.101402

[2]Thresholds and weights are from Pejaver et al. (2022). Calibration of computational tools for missense variant pathogenicity classification and ClinGen recommendations for PP3/BP4 criteria. Am J Hum Genet 109, 2163–2177. doi: 10.1016/j.ajhg.2022.10.013
